# Interfacial Electrochemical Lithiation and Dissolution Mechanisms at a Sulfurized Polyacrylonitrile Cathode Surface

**DOI:** 10.1021/acsenergylett.3c02757

**Published:** 2024-02-05

**Authors:** Dacheng Kuai, Shen Wang, Saul Perez-Beltran, Sicen Yu, Gerard A. Real, Ping Liu, Perla B. Balbuena

**Affiliations:** † Department of Chemical Engineering, Texas A&M University, College Station, Texas 77843, United States; # Department of Chemistry, Texas A&M University, College Station, Texas 77843, United States; § Department of Materials Science and Engineering, Texas A&M University, College Station, Texas 77843, United States; ∥ Department of Nanoengineering, University of California, San Diego, La Jolla, California 92093, United States; ⊥ Materials Science and Engineering Program, University of California, San Diego, La Jolla, California 92093, United States

## Abstract

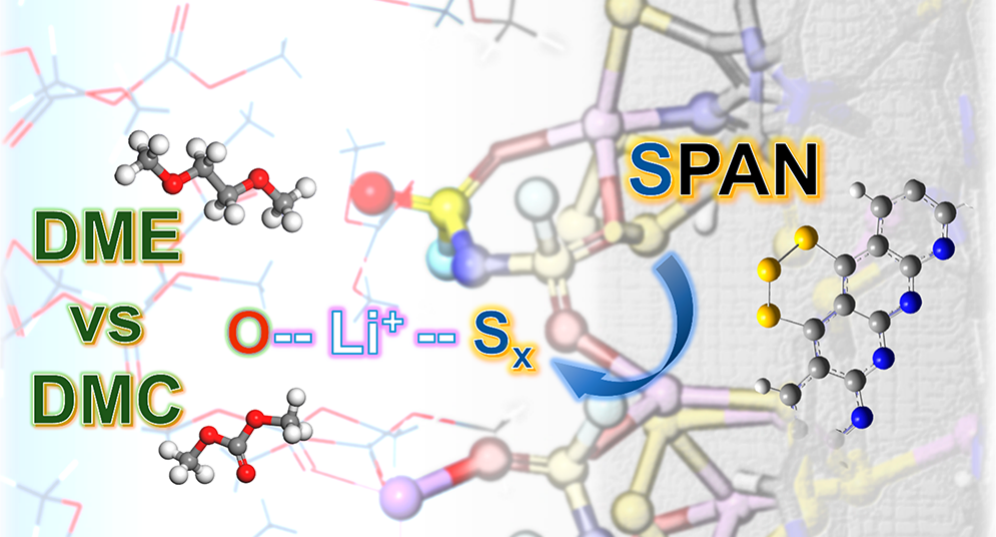

Advances in sulfurized-polyacrylonitrile (SPAN)-based cathode materials promise safer and more efficient lithium–sulfur (Li-S) battery performance. To elucidate electrolyte–cathode interfacial electrochemistry and polysulfide (PS) dissolution, we emulate discharge SPAN reactions via *ab initio* molecular dynamics (AIMD) simulations. Plausible structures and their lithiation profiles are cross-validated via Raman/IR spectroscopy and density functional theory (DFT). Lithium bis(fluorosulfonyl)imide (LiFSI) plays versatile roles in the Li-SPAN cell electrochemistry, primarily as the major source in forming the cathode–electrolyte interphase (CEI), further verified via X-ray photoelectron spectroscopy and AIMD. Besides being a charge carrier and CEI composer, LiFSI mediates the PS generation processes in SPAN electrochemical lithiation. Analysis of AIMD trajectories during progressive lithiation reveals that, compared to carbonates, ether solvents enable stronger solvation and chemical stabilization for both salt and SPAN structures. Differentiated CEI formation and electrochemical lithiation decomposition pathways and products are profoundly associated with the intrinsic nature of lithium bonding with oxygen and sulfur.

Within the cohort of next-generation energy storage devices, lithium–sulfur (Li-S) batteries promise superior cost economy and theoretical energy density.^1,2^ Many investigations focus on developing sulfur-based cathode materials by elevating their electronic and ionic conductivity and cell energy density. Sulfur is intrinsically a good electronic insulator. High specific capacity is positively associated with sulfur loading, but difficulties in transport and irreversible structural changes during cycling may compromise the electrochemical performance. One strategy for balancing the two major cell property criteria is to bind sulfur atoms onto a conductive matrix. Carbon-based materials have been developed for this purpose, including sulfurized carbon nanotubes,^3^ graphene,^4^ and polyacrylonitrile (PAN).^5^ The emerging sulfurized-polyacrylonitrile (SPAN) materials demonstrate excellent cycling performance and reversibility, although limited information about structures and redox pathways hinders larger scale applications.^5−7^

Cross-talk of active species results in anode/cathode deactivation and capacity fading in Li-S batteries.^8^ Such a shuttling effect can be alleviated by tuning the electrolyte and suppressing polysulfide (PS) dissolution. Ether-based electrolytes usually have a higher sulfur utilization rate,^9^ while carbonate-based ones commonly have better cycling stabilities.^10^ A mechanistic understanding of the solvents’ roles in representative chemical events during SPAN cycling is pivotal in rational engineering of battery materials. Density functional theory (DFT)-based molecular modeling offers in-depth electronic structure and potential energy surface information.^11,12^ Elucidating the explicit solvent–intermediate interactions and interfacial effects beyond the continuous solvent approaches are vital to predicting the corresponding reaction behaviors and kinetics.^13−15^ In the SPAN case, the C/N–S and S–S cleavage/formation reactions usually take place at the electrolyte–cathode interface, where the electrolyte solvent, salt, and PAN backbone may simultaneously contribute to the reaction.

Bonding categories involved in the SPAN electrochemical lithiation/delithiation are typically diverse,^16^ and many efforts have been made to enhance the fundamental understanding of the complex interfacial electrochemical reaction mechanisms.^17^ In addition to PS cleavage and reattachment, the PAN backbone experiences structural and chemical rearrangements during cycling.^18^ To facilitate the atomistic understanding of sulfur cathodes, classical theoretical approaches^19−21^ have proved useful for describing phenomena such as PAN vulcanization at an atomistic level.^22^ The SPAN lithiation^23^ and solvation effects in linear and cyclic carbonate electrolytes^24^ were further studied at the *ab initio* level of theory.

Cathode–electrolyte interphase (CEI) layers play an essential role in tuning the electrochemical performance of Li-S battery systems.^25^ Carbonate species from solvent decomposition and polymer cathode additives protect the cathode from severe sulfur dissolution^26^ and form an electrostatically repulsive barrier.^27^ Other electrolyte formulations have enabled reduced shuttling effects, long Li-SPAN cell lifespan, and wide temperature tolerance.^28^ The LiFSI salt not only serves as a charge carrier but also easily dissociates in redox reactions, contributing to the solid–electrolyte interphase (SEI)^29^ and CEI.^30^

Herein we identify plausible SPAN structures obtained in vulcanization processes and elucidate their corresponding lithiation/reduction mechanisms. We compare the roles of carbonate and ether electrolytes during the interfacial lithiation of SPAN structures and explore the unique role of LiFSI in the chemistry at the SPAN–electrolyte interface. We obtain an overview of the lithiation reactions and solvation effects as a function of SPAN structural features and site-dependent lithiation energetics and further assess the interactions between electrolyte species and reaction intermediates during electrochemical lithiation through *ab initio* molecular dynamics (AIMD) simulations incorporating detailed electronic structure analysis of key chemical events.

Many proposed SPAN structures have been probed with characterization techniques including solid-state nuclear magnetic resonance (ss-NMR),^31,32^ X-ray photoelectron spectroscopy (XPS),^32^ Fourier transform infrared spectroscopy (FTIR), and X-ray diffraction (XRD),^33,32^ summarized by Zhao et al.^5^ To estimate and verify the thermodynamic favorability of different sulfur chain types, we compute the thermochemistry of forming designated C–S and N–S bonds. Compared to C–C bonding, the shorter N–C distance leads to the *C*_2*v*_ symmetry of the pyridine units in PAN backbone, which makes it challenging to optimize the SPAN structure in periodic boundary conditions without fully relaxing the ring tension. Here we select a structure with four pyridine units to serve as the PAN backbone in estimating the vulcanization energies. To eliminate the formation energy variations in different sulfur allotropes and to make unbiased comparisons among species, Figure 1 shows the average electronic energy of each S atom in the S_8_ molecule as the S_*n*_ energetics on the product side when calculating the vulcanization energetics. Note that this approach underestimates the thermodynamic favorability in many cases because smaller and more active sulfur molecules are readily available under the high vulcanization temperature.^34^ For instance, when the S_2_ molecule is used as the starting molecule for obtaining the bridge–CN structure, enthalpy changes for “parallel” (para) and “perpendicular” (perp) configurations become −87.66 and −91.68 kcal/mol, and the free energy changes at 723 K reach −10.29 and −14.36 kcal/mol, respectively.

**Figure 1:**
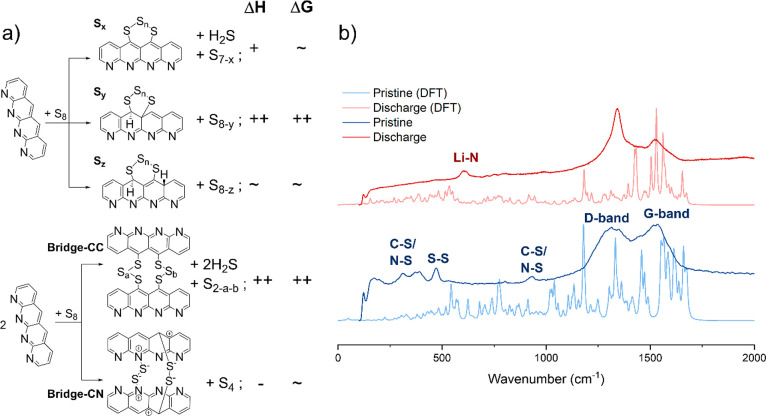
SPAN structural studies via (a) DFT calculations of vulcanization reaction energetics at 723 K and (b) Raman spectra of DFT-verified SPAN models and experimental measurements before and after electrochemical lithiation. The “+” and “++” signs in (a) suggest positive values below and above 25 kcal/mol, respectively; “–” suggests negative values larger than −25 kcal/mol; “∼” indicates positive and negative values that vary depending on structures in the same category and reference starting materials. The atomic charge signs only serve the purpose of visualization and do not reflect the actual charge states. Detailed thermodynamic and electronic structure information is available in Figure S1. In (b), vibrations on PAN backbones such as C–H and C–N are not labeled for clarity.

As suggested for S–C(sp^2^), S–C(sp^3^), and S–N formation, the even-numbered sulfur chains are thermodynamically more stable than the odd-numbered ones, not only by themselves (see Figure S1 for details) but also when attached to PAN backbones. The energy barriers for the transition between S_*y*_- and S_*z*_-type structures were calculated to be as high as 81.42 kcal/mol when an S_4_ chain is attached, suggesting that such transformation is highly unlikely. Meanwhile, when attempting to optimize the structure of bridging sulfur chains that connect PAN on nitrogen sites, local minima were always located when the S–C(sp^3^) covalent bonds are formed instead (see Figure S2 for details).

Spectroscopic methods are powerful in resolving bonding information in solid-state materials. To further understand and validate the SPAN structural models and discharge profiles derived from the first-principles calculations, we study the Raman and infrared (IR) spectroscopy of pristine SPAN and the evolutions due to the first discharge process. Computed structures are summed and normalized from two major plausible SPAN molecular models: “bridging” S_*x*_-PAN (*x* = 2, 3) and “single-sided” S_3_-PAN. Most of the Raman/IR-active vibrations have good agreement with the detected peaks from experiment and empirical assignments (Figure 1b). The overestimated H atomic ratio in saturating the peripheral PAN atoms, together with the presumed molar ratios of all proposed SPAN structures, is responsible for the wavenumber and intensity displacements between simulation and measurement. After complete lithiation, an observable increase in the intensity ratio of the D-band to G-band (*I*_d_:*I*_g_) signals a reduced degree of structural order following lithiation processes. The spectra display characteristic D-band and G-band features within the 1000–1500 cm^–1^ range. Notably, in the region below 1000 cm^–1^, a series of peaks associated with C–S, N–S, and S–S bonds are observed. Regarding the Raman spectra of pristine SPAN, there are uncertainties in peak assignments below 1000 cm^–1^.^18,35^ Peaks around 900 cm^–1^ were assigned to either a C–S or S–S vibrational mode. Recent studies have also suggested an N–S vibrational mode occupying this same spectral position. All these peaks diminish upon discharge completion, suggesting either cleavage of these bonds or a transition into a lithiated form with a diminished Raman scattering cross-section. In addition, a novel peak emerging near 600 cm^–1^ after discharge was attributed to Li–N vibrations instead of the C–S lithium-related compound discussed in earlier reports.^36−38^ Assisted by the DFT predictions, all peaks are reassigned accordingly with more details in Figure S3, in addition to the FTIR spectra shown in Figure S4.

In this study, we explored the impact of di- and trisulfide bridging on free energy variations in SPAN. To provide experimental support, we conducted time-of-flight secondary ion mass spectrometry (ToF-SIMS) analysis. The findings, presented in Figure S5, reveal the presence of S^–^, S_2_^–^, and S_3_^–^ ions (*m*/*z* 32, 64, and 96) in the synthesized SPAN. Notably, ions representing longer sulfur chains, such as S_4_^–^ (*m*/*z* 128) and S_5_^–^ (*m*/*z* 160), were almost undetectable. This suggests a minimal presence of longer sulfur chains (S_*x*_, where *x* > 3) in SPAN. Therefore, we primarily focused on the lithiation behaviors of disulfide and trisulfide structures. The first reaction step initiates from electron uptake to serve as a comparison against lithiation via Li^+^. The following steps are calculated by continuously introducing lithium atoms to the designated sites, as indicated in Figure 2 (details available in Figures S6 and S7). According to the free energy profiles, solvation by continuum solvent manipulates the early stage of the lithiation reactions. Polarities and charge distributions of reduced intermediates vary greatly in these steps, resulting in significant changes in solvation heat. The “linear” SPAN in Figure 2a has an S_3_ chain bonded on the carbon side of an H-saturated 4-unit PAN oligomer. In such a structure, the electron uptake and Li^+^ coordination steps in the continuum solvation model experience significant energy uphill and downhill, while electrochemical relaxation is less irregular in gas-phase processes. A similar phenomenon was observed in other SPAN structures where PS is bonded to sp^3^ carbon atoms, as indicated in Figure S8. This suggests that the solvation environment could be critical to electrochemical cycling in C–PS-bonded “linear” SPAN structures and may result in energy barriers in the early discharge processes.

**Figure 2:**
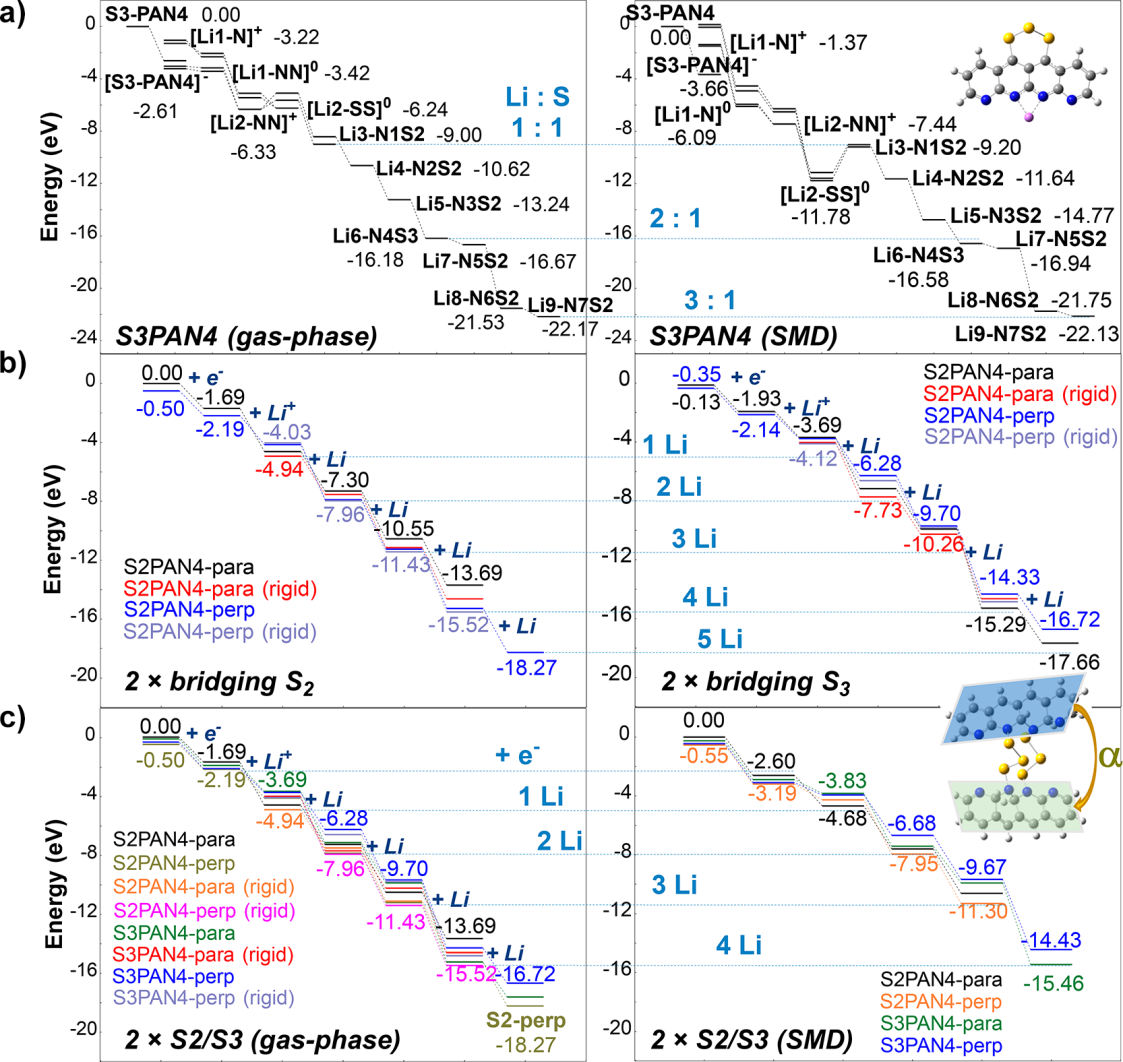
DFT-calculated lithiation free energy profiles of different PS chain categories bonded to 4 units of pyridine oligomer(s) backbones. (a) Free energy energetics of a single-sided SPAN molecular model computed in the gas phase and via the SMD model. (b) Lithiation free energy profile comparison between S_2_-bridged and S_3_-bridged molecular models. (c) Performance variation from gas phase to implicit solvation. Each color in parts b and c represents the corresponding bridging configuration (S_2/3_ with “parallel” (para) or “perpendicular” (perp) backbone relative angles). Dashed lines facilitate comparison at the equivalent lithiation stages with respect to the left-side data points.

Besides the PS anchored on single-sided C(sp^2^), bridging sulfur chains between PANs also have considerable abundance. Depending on the PS availability and relative distances between two PAN backbones, different varieties of bridging PSs are likely to exist in freshly synthesized SPAN and after electrochemical cycles. We analyze differences between disulfide- and trisulfide-bridged SPAN structures and solvation effects. During charging/discharging cycles, the PAN framework could either remain stationary due to the rigid surrounding space or twist to fit for lower steric hindrance. Therefore, we optimized geometries with frozen far-end carbons as well as the ones without constraints shown in Figure 2c with color coding. The lithiation energetics of S_2_- and S_3_-bridged SPANs are generally consistent with each other, except that the S_2_ case has more negative free energy changes than the S_3_ one by 0.8–1.2 kcal/mol in the first and third lithiation steps. Introducing solvation to PS-bridged SPANs does not induce significant free energy variations.

Due to the electronegative nature of S and O, PS dissolution is usually mediated through Li^+^ coordination, as Li is the only abundant cationic species in Li-S battery systems. When clustered with PSs, the electronic structures and binding energetics of [Li-solvent]^+^ are profoundly altered. With representative [Li_2_S_*x*_-solvent] cluster configurations, Figure 3 demonstrates how binding energies between Li_2_S_*x*_ and the solvent (Li^+^–O_solvent_ bond formation energy) evolve with respect to the sulfur chain length in different solvation environments. The dielectric constant is one of the critical parameters in presenting the solvation strength, especially when evaluating with the continuum solvation models.^39^ We selected dielectric constants of 4.24, 7.43, and 109 to model environments with low to high solvation effects, influenced by the salt effect on the solvent’s dielectric properties. The low constants (4.24 and 7.43) represent solvents like diethyl ether and tetrahydrofuran, while the highest constant (109) reflects the highly polar environment of an ethylene carbonate–vinylene carbonate solvent, broadening the model’s applicability across diverse electrolyte systems. It is important to note that these values represent average conditions in our implicit solvation model rather than specific solvents or electrolytes. Being statistically 95% confident, sulfur chain length *x* is positively correlated with the DOL-Li_2_S_*x*_ binding free energies, but not in the other two solvents; the dielectric constant has a negative contribution toward the binding energies between DMC and Li_2_S_*x*_, while there is no significant impact on DME or DOL. Clear boundaries between Li and S atoms in electronic Laplacian isosurfaces (blue glassy structure in Figure 3) reveal bonding ionicity between Li^+^ and S_*x*_^2–^. In contrast, despite poor electronic Li and O_(solvent)_ density overlap in all clusters, positive Laplacian values in their bridging spaces suggest semi-covalent Li^+^–O_(solvent)_ bonding. In Li^+^-mediated solvent–SPAN interactions, the PSs bonded on the PAN backbone display consistent Li^+^–S or Li^+^–O_(solvent)_ bonding nature, as discussed. Therefore, it is reasonable to treat [Li^+^-solvent] as an ionic cluster that dissolves PSs via electrostatic forces.

**Figure 3:**
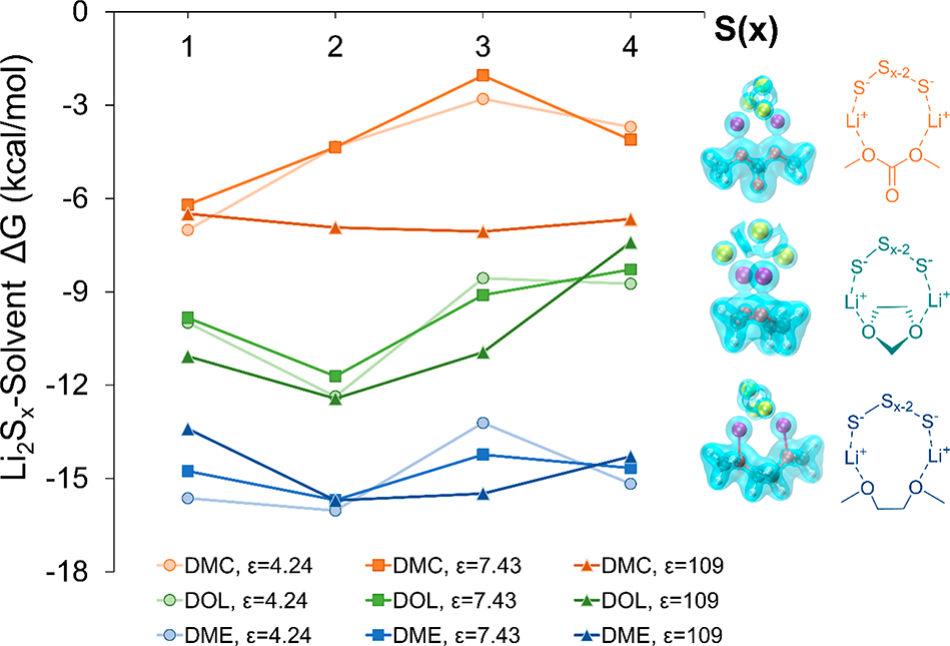
Binding free energy between Li_2_S_*x*_ and solvent molecule evolution with respect to the S_*x*_ chain length in different implicit solvation systems. The right-side figures denote the electronic Laplacian isosurfaces (isovalue = 0.09) of the corresponding structures (*x* = 3).

The simplified molecular model reflects the performance of SPAN’s individual sulfur chain categories, while coexisting PS varieties induce higher complexity in the SPAN electronic structures as well as in the corresponding lithiation reaction profiles. AIMD simulations (Figure 4) further help to elucidate the electrolytes’ roles in the SPAN lithiation reaction pathways and the dissolution of generated PS species at interfacial (∼10 nm) scales. We compare two diluted electrolyte systems: DMC-solvated 1.2 M LiFSI and DME-solvated 1.0 M LiFSI. DME’s stronger PS dissolution capability compared to DMC profoundly affects SPAN’s electrochemical lithiation reaction mechanisms. The pristine SPAN structure was retrieved from previous work.^22^ According to Figure 4a,b, the electronic spin density is primarily distributed along the PAN backbone, suggesting that the high-spin-state electrons mainly exist among the carbon and nitrogen atoms with delocalized electrons instead of staying on the PSs. These light atoms with high-spin electrons have higher priority to couple with external electrons than sulfur atoms when discharging. This is also associated with the irreversible capacity loss due to an aromaticity decrease after the first discharge cycle (Figure S9).^18^ During the lithiation process, the monosulfide species dissolved in the electrolyte continuously increase for both solvents; in the meantime, the counts of disulfides and trisulfides in the electrolyte increase when the PSs dissociate from the PAN backbone and oscillate during the dynamic electrochemical lithiation. Figure 4c shows a clear dependence of the PS dissolution on the chain length, nature of the solvent, and Li/S ratio. Note that not all disulfide structures were eventually converted to monosulfide. As stated in the previous binding energy analysis, the high solubility of PS in DME results in a larger population of intermediate Li_*x*_S_2_^*x*–2^ and Li_*x*_S_3_^*x*–2^ species being dissolved compared with that in DMC. The average PS chain length evolution profile offers an overview of the C/N–S and S–S bond cleavage progress during lithiation. A shorter average PS chain length suggests a higher bond cleavage level induced by lithiation. DMC induces less dissolution and stabilization for PSs, thus leading to phenomena including (1) PS rearrangement events reflected in fluctuations in Figure 4d and (2) slower and less complete C/N/S–S bond cleavages in high lithiation stages.

**Figure 4:**
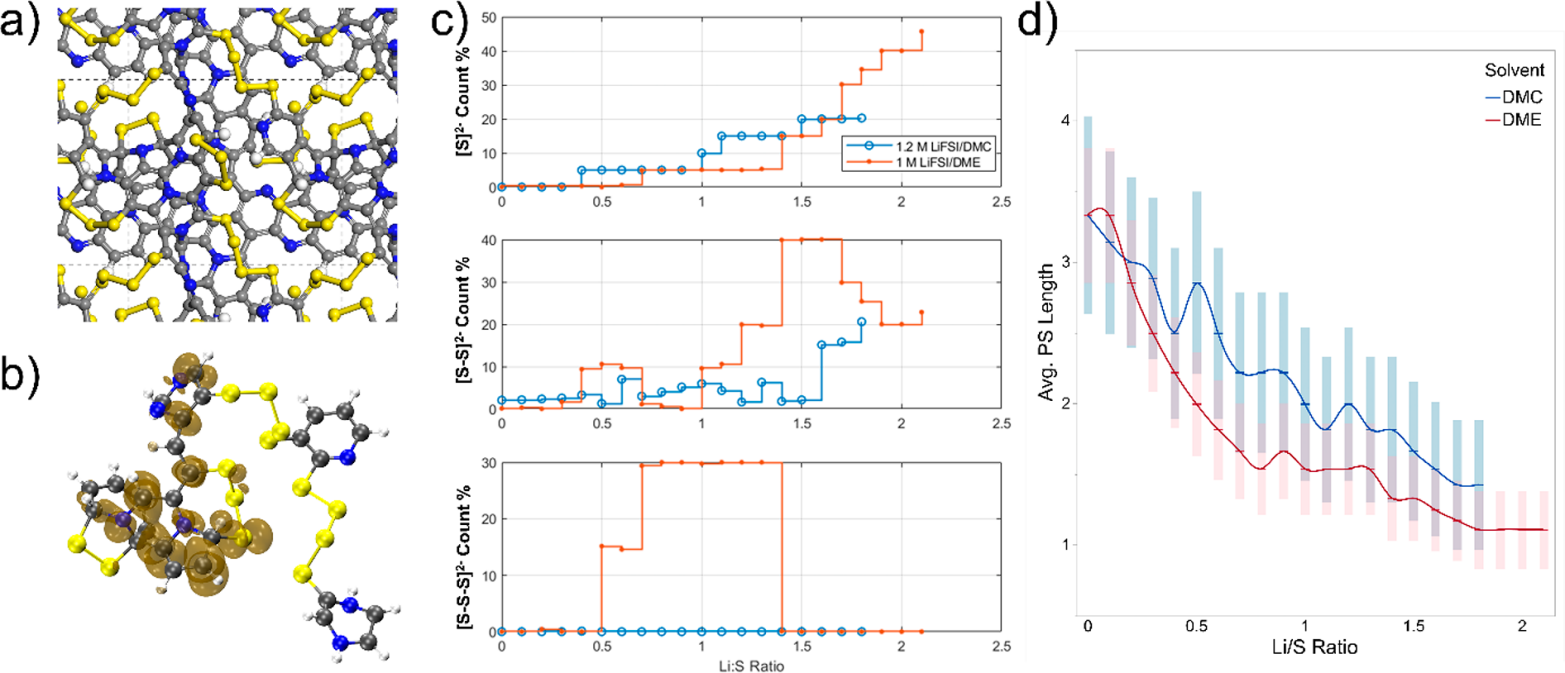
Overview of the SPAN structure and electrochemical lithiation reactions from AIMD. (a) Top view of pristine SPAN structure shown in repeated cell units, with dashed lines indicating the cell margins. (b) Electronic spin density distribution of the “upper layer” moiety. The number of hydrogen atoms on peripheral C/N atoms in the molecular model is determined by the pristine bonding conditions. (c) PS count percentage by lengths in DMC (blue circle) and DME (red dot) liquid electrolyte phases as a function of discharge Li/S ratio. (d) Average PS chain length (number of units) evolution profile during lithiation. The shadow areas indicate the corresponding 90% confidence intervals.

As a major source of CEI composition, LiFSI decomposition on the SPAN CEI is confirmed from prior reports^17,18^ and further elucidated in this work via XPS. Aside from the previously observed N(1s) and S(2p) signals,^18^ F^–^ ion and S–F bonding signals were detected on the SPAN due to LiFSI defluorination and dissociation, as Figure S10 denotes. According to the AIMD simulations of the CEI-forming process, solvation dynamics in carbonate and ether electrolyte lead not only to differentiated PS behavior during lithiation but also to LiFSI degradation pathway variation, as suggested in Figures S11 and S12. In the DMC electrolyte system, N–S cleavage has higher priority than defluorination caused by S–F breaking, while DME solvation has an opposite result. Within the AIMD ensemble, LiFSI degradation generates a more diverse group of product fragments, in a faster manner, in the carbonate electrolyte than in DME. Within the picosecond simulation time scale, for the first time, we observe that only in the DMC-based electrolyte system, after the SO_2_F^–^ and NSO_2_F^2–^ fragments are generated by N–S cleavage, the SO_2_^2–^ fragment from further SO_2_F^–^ degradation can covalently bond with PS on the SPAN cathode surface. Previously the reduced SO_2_ species were recognized in enhancing the graphite anode’s performance.^40^ This process allows post-synthesis surface modification to the SPAN structure during charging/discharging cycles, resulting in effects similar to those reported by Ein-Eli et al.^40^ Within the AIMD time frame, the remaining fragments of FSI degradation have a higher preference to adsorb on the SPAN surface than diffusing in electrolyte, thus facilitating CEI formation, with solvent-dependent compositions. This also explains why predominant XPS signals from LiFSI fragments can be detected after the cathode residual-cleaning protocols.

Significant chemical events observed in AIMD simulations enhance our understanding of lithiation reaction mechanisms. We retrieve partial geometries of representative chemical events, focusing on PS cleavage and dissolution processes at the SPAN–electrolyte interface. In the DME-solvated system, PSs are well stabilized and experience consecutive S–S cleavages. Figure 5 shows that, for a Li:S ratio of 1:1, an S_4_ radical chain which has one side bonded to the PAN backbone heterolytically cleaves into an S_3_ and an S atom. The S_3_ structure coordinates with multiple Li^+^ and dissolves in the electrolyte, while S remains bonded to the PAN structure. Beyond its common role in serving as an ionic charge carrier, FSI^–^ is constantly observed mediating the electrochemical lithiation and facilitating the solvation shell formation for PSs according to our AIMD simulations. The bonding natures, including semi-covalency and ionicity, of Li^+^-O/S in the whole configuration remain consistent with the ones discussed for Li_2_S_*x*_-solvent clusters. Thus, FSI^–^ together with DME coordinates with Li^+^ to act as an entity when participating in the PS reductive evolution (Figure 5a).

**Figure 5:**
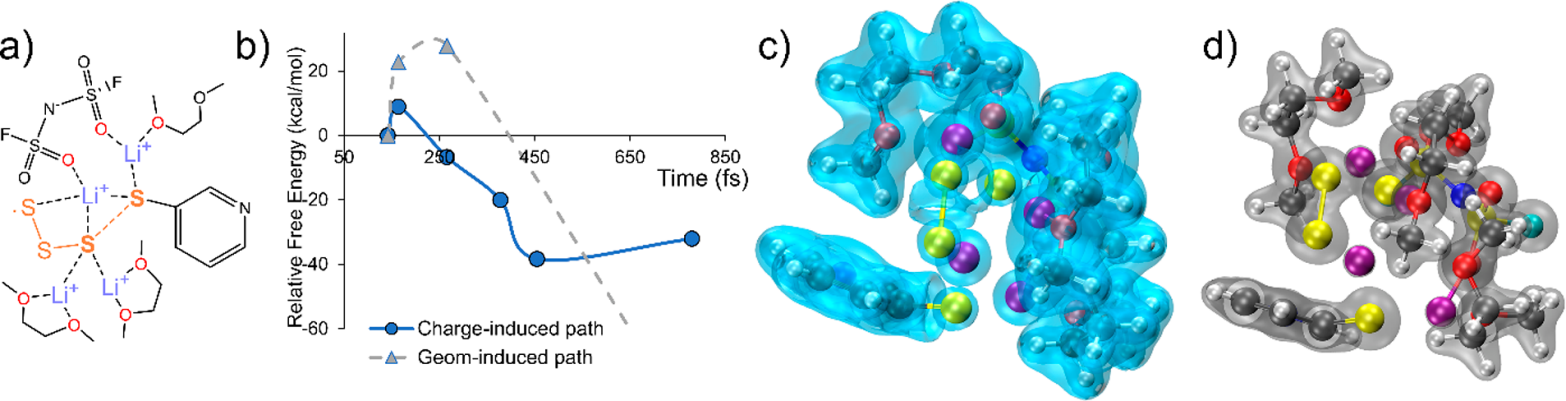
Electronic analysis of DME-LiFSI-mediated S–S cleavage. (a) General configuration of the dissociation event. Dashed lines between S atoms indicate broken bonds. (b) Free energy profiles comparison between charge-transfer and geometry-induced decomposition pathways. Electronic Laplacian (c) and electron density (d) isosurfaces of the configuration at 780 fs, after the cleavage is accomplished (isovalue = 0.09) in the charge-induced path.

According to the Bader charge analysis of the AIMD trajectory,^41^ the whole Li-FSI-DME-PS-PAN complex takes up one electron throughout the S–S cleavage process. To clarify the triggering factors in this reaction, we performed single-point calculations based on two paths: (1) electron uptake takes place first, which leads to the increase in the S–S distance (curve with blue circles in Figure 5b), and (2) geometry variations promote the charge transfer to initiate (curve with gray triangles in Figure 5b). The charge-transfer-induced pathway experiences only 8.87 kcal/mol free energy uphill, while the geometry-variation-induced one requires overcoming a 27.83 kcal/mol barrier, which is significantly less likely to happen in a picosecond process. After the S–S cleavage, the dissociated S atoms do not have overlapping electronic density or Laplacian (Figure 5c,d), while the Li–O and Li–S coordinations retain their semi-covalent and ionic properties, respectively. Therefore, our evidence suggests that the major cause of PS dissociation is electron transfer into the SPAN cathode materials, assisted by the electrolyte-mediated Li^+^ coordination.

Figures S13 and S14 provide in-depth information regarding the charge distribution variations during SPAN’s electrochemical lithiation. Similar to the PS length distributions, atomic charges have more vigorous fluctuations in DMC electrolyte than in DME. Unlike the presumed scenarios in which S_SPAN_ atoms take the external charges by transforming into lithiated PS, according to our Bader charge analysis, sulfur in general receives only approximately 40% of the external charges, while the remaining 60% are transferred to the C on the PAN backbone after Li:S approaches 2:1. This is associated with the electronic structures reflected in Figure 4b, where most of the high-spin electronic density is accumulated on C and N atoms that have higher priority in coupling with electrons during the discharging process. The later-stage electrochemical lithiation mainly leads to distortions in the PAN electronic structures. This agrees well with the discharge/charge curve determined by Wang et al., indicating that the irreversible capacity loss is due to the transformation into non-aromatic structures.^18^ Interestingly, upon reaching a Li:S ratio of 1:1, more than 65% of the charge transferred to S, which corresponds to the moment when the largest amount of trisulfide species left the PAN backbone. This suggests that most of the sulfur reduction takes place in the first half of the discharging process.

In conclusion, based on DFT computations, we found that the PSs binding with the carbon atoms with sp^2^ hybridization on a single side of the PAN backbone, as well as the ones bridging two neighbor PAN chains, are plausible structures in SPAN. The corresponding Raman and IR spectroscopic measurements further confirmed the proposed structures. The electrochemical lithiation of the single-sided SPAN structures has a stronger tendency to be impacted by the solvation environment than the bridging PS case. Via first-principles AIMD approaches, we also analyzed the electrolyte’s dissolution and its influence on the first charging cycle. The PS length and dielectric constant have statistical correlations with the [Li_2_S_*x*_-solvent] binding energies in certain solvent systems; however, they do not alter the solvation strength order: linear carbonate (DMC) < cyclic ether (DOL) < linear ether (DME). DMC’s weak solvation effects result in diminishing PS stabilization during the reduction reactions. This phenomenon is evidenced by the shorter average PS chain lengths and altered charge distributions observed in our study. Conversely, the robust solvation associated with DME tends to promote significant PS shuttling and potential anode degradation in subsequent charge/discharge cycles. Strategies to mitigate these adverse effects stemming from the strong dissolution of DME include (1) functionalizing, especially by fluorinating, the linear ether structures to tune PS solubility,^42^ (2) employing a higher proportion of cyclic ethers and carbonates in the electrolyte composition to balance solvation strength, and (3) developing advanced electrolyte additives that can more effectively control solvation dynamics and PS behavior while keeping Li metal anode stability as another key target.

When we further resolved the AIMD trajectories of SPAN lithiation, we found that LiFSI’s contribution in Li-SPAN chemistry goes beyond its roles as charge carrier and anode SEI source component. The interfacial LiFSI fragmentation on the SPAN surface is confirmed from both AIMD simulation and XPS signals. The degradation product fragments on the cathode become part of CEI, and the SO_2_^2–^ could further bond with PS in the SPAN system by forming S–S bonds. Furthermore, FSI^–^ mediates the S–S cleavages in PS through semi-covalent Li^+^-O coordination and Li^+^-S electrostatic interactions, allowing electron-transfer-induced pathways to proceed with a low energy barrier. The insights into the versatile roles of the LiFSI-DME system bring new rationale in next-generation Li-SPAN electrolyte design: the presence of dual oxygens in DME, with optimal distances for chelating Li^+^, is a positive factor to maintain. Based on the DME framework, the electrolyte solvent engineering direction is to reduce the PS dissolution strength to a moderate level and further expand the voltage tolerance window. Fluorination has been proven to be a promising strategy for these purposes in creating a desired SEI on LMB anodes.^42,43^ However, how the DME with different degrees of fluorination would interact with SPAN cathode materials with LiFSI remains unresolved and could be a potential direction to explore both experimentally and via multiscale simulations.

## Experimental Details

DFT analysis was performed at the B3LYP/aug-cc-pVDZ level of theory based on the Gaussian 16 package. Grimme DFT-D3 dispersion correction was employed in all calculations.^44^ All structures were optimized to their energy local minimum without an imaginary vibrational mode. Implicit solvation effects were evaluated based on the SMD model.^45^ The electronic density and Laplacian isosurfaces were generated via the Multiwfn package^46^ and visualized with VMD.^47^

The initial configuration of AIMD simulations was obtained by packing electrolyte solvents and LiFSI with SPAN structure^48,49^ in a slab of 35.00 × 13.06 × 11.31 Å^3^ dimensions based on the Amorphous Cell Packing module in Materials Studio (v8.0). Two electrolytes, i.e., DMC- and DME-solvated LiFSI systems, were studied. The liquid-phase density was verified to be consistent with the available data. The AIMD simulations were performed via Vienna Ab initio Simulation Package (VASP) 5.4.4.^50^ The Perdew–Burke–Ernzerhof (PBE) functional was used to describe the electron exchange and correlation energies within the generalized gradient approximation (GGA).^51^ Electron–ion interactions were considered within the projector augmented wave (PAW) pseudopotentials^48,49,52,53^ in a slab with dimensions of 35.00 × 13.06 × 11.31 Å^3^ based on the Amorphous Cell Packing module in Materials Studio (v8.0). Each batch of the AIMD based on the NVT ensemble was run for 10 ps with a 1.2 fs time step. To emulate the battery discharging process, 2 Li atoms were introduced within 1.5 Å radial distance with respect to N atoms in the final frame to initiate the next simulation cycle. The lithiation cycles end when the Li:S ratio approaches 2:1. The DMC-solvated simulation system ceased when 36 “external” Li atoms were introduced, and the DME case lasted until 42 Li atoms were introduced. The atomic Bader charges were analyzed based on the grid-based method developed by Henkelman et al.^41^ The Open Visualization Tool (OVITO 3.7.5)^54^ was employed in post-simulation analysis and visualization.

## Supporting Information Available

Vulcanization reaction energetics (Figure S1); electron density isosurfaces (Figure S2); DFT-predicted Raman spectra (Figure S3); experimental and DFT-predicted FTIR spectra (Figure S4); ToF-SIMS spectrum of SPAN (Figure S5); stepwise SPAN geometry change in lithiation (Figure S6); lithiation-led geometry change in bridging SPAN configurations (Figure S7); electrochemical reduction free energies (Figure S8); discharge/charge profiles (Figure S9); high-resolution XPS analysis (Figure S10); FSI decomposition pathways (Figure S11); S_2_–SO_2_ formation (Figure S12); charge evolutions during SPAN lithiation (Figures S13 and S14); description of experimental methods (PDF)
