# Supplementary material for: Interfacial Electrochemical Lithiation and Dissolution Mechanisms at a Sulfurized Polyacrylonitrile Cathode Surface
Source: ACS Energy Lett. 2024 Feb 5;9(3):810–8. doi: 10.1021/acsenergylett.3c02757 (PMC10928710; doi:10.1021/acsenergylett.3c02757)
Supplement: Supplementary file 1 — nz3c02757_si_001.pdf [file nz3c02757_si_001.pdf]

Supporting Information for:

Interfacial Electrochemical Lithiation and Dissolution Mechanisms at Sulfurized Polyacrylonitrile Cathode Surface

Dacheng Kuai[a,b,‡], *Shen Wang*[d,‡], *Saul Perez-Beltran*[a], *Sicen Yu*[d], *Gerard A. Real*[a], *Ping Liu*[d,e *], *and Perla B. Balbuena*[a,b,c *].

a. Department of Chemical Engineering, Texas A&M University, College Station, Texas 77843, USA

b. Department of Chemistry, Texas A&M University, College Station, Texas 77843, USA

c. Department of Materials Science and Engineering, Texas A&M University, College Station, Texas 77843, USA

d. Department of Nanoengineering, University of California, San Diego, La Jolla, California 92093, United States

e. Materials Science and Engineering Program, University of California, San Diego, La Jolla, California 92093, United States

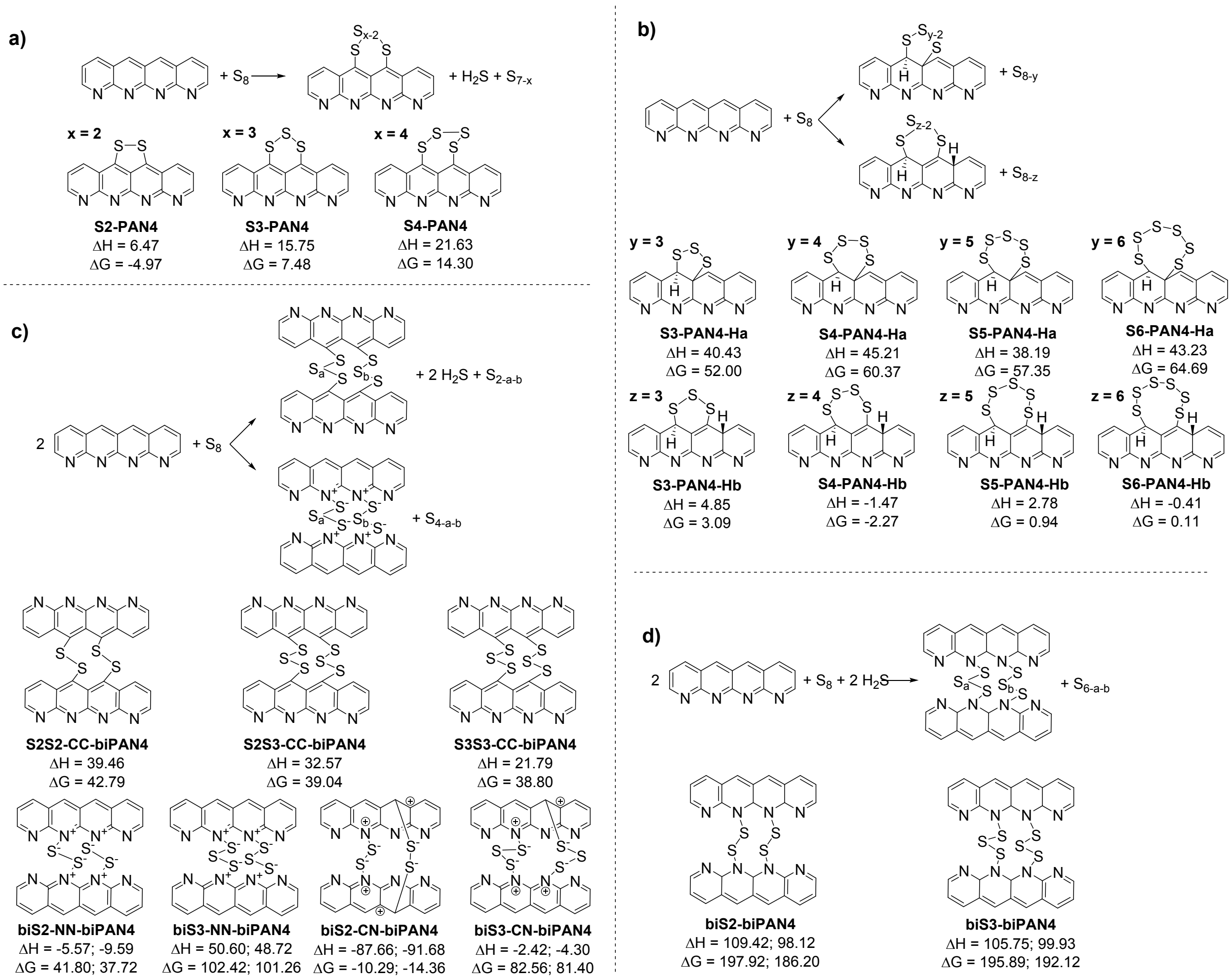

Figure S1. Vulcanization reaction energetics (in units of kcal/mol) computed at B3LYP/aug-CC-pVDZ level of theory. The enthalpy values are obtained at the default temperature of 298.15 K, and the free energy are converted to 723.15 K for the convenience of comparison at synthesis conditions. The structures with two values in enthalpy and free energy changes indicate the configurations with relative PAN positions of “parallel” and “perpendicular”, respectively.

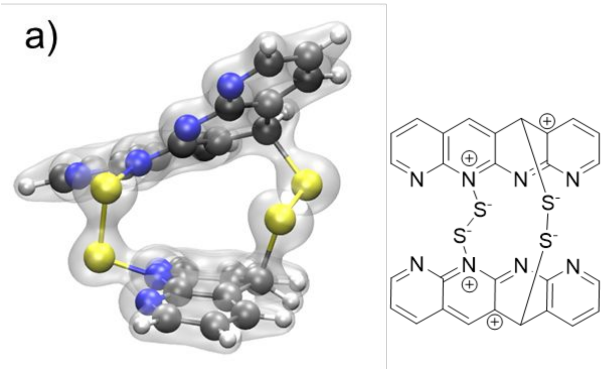

Figure S2. The electron density isosurfaces (iso-value = 0.1) of two representative PS-bridging structures, biS2-NN-biPAN4 (a) and biS3-NN-biPAN4 (b), respectively. The + and – charge symbols on the chemical structures do not reflect the real charge distribution. The N-S and C-S bonding both show covalent properties.

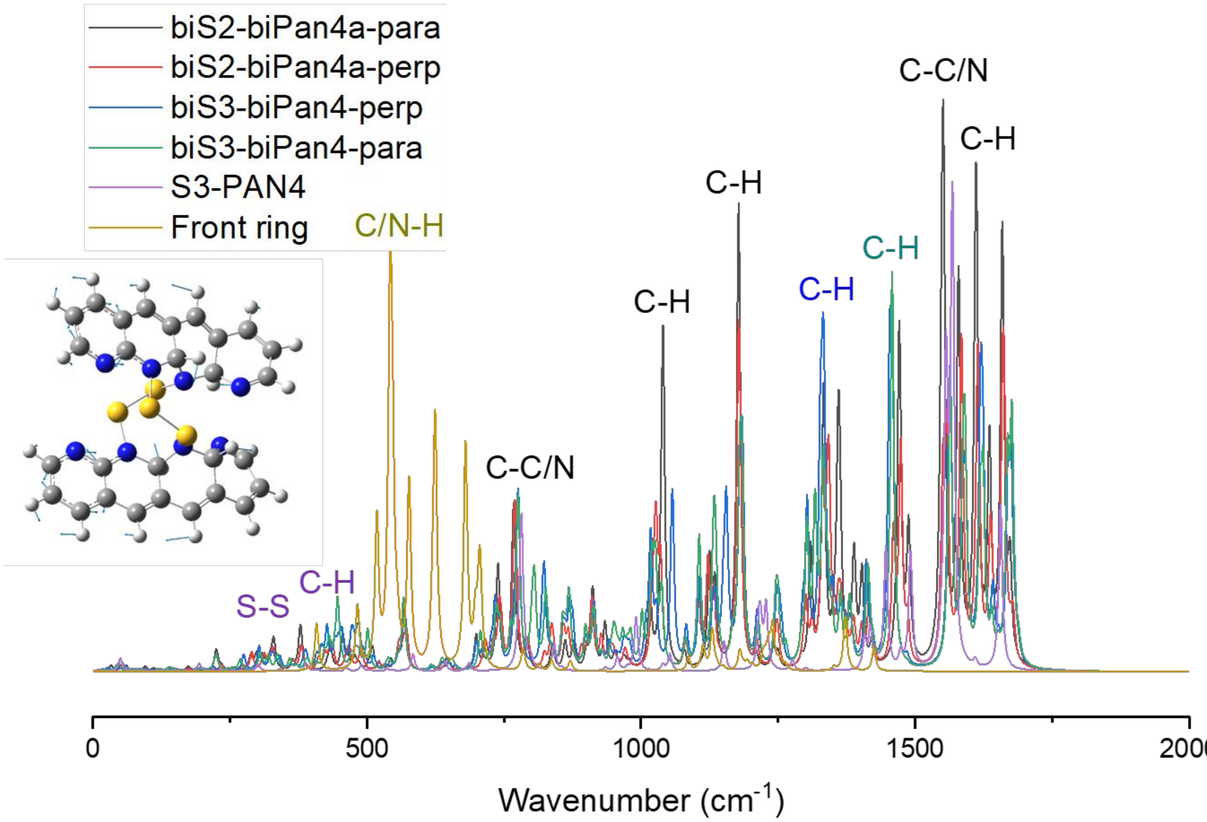

Figure S3. Predicted Raman spectra of pristine SPAN molecular models and the visualization example of IR and Raman identification with the biS2-biPAN4a(para) vibration at 1333 cm^{-1} (blue arrows indicate the atomic motions). Note that the “Front ring” is the molecular fragment of the periodic SPAN structure applied in AIMD.

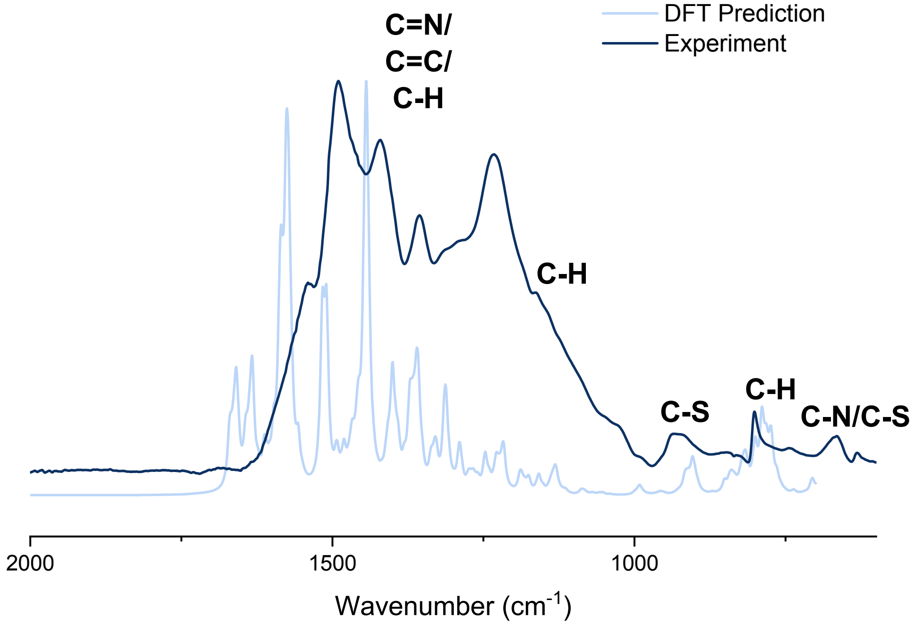

Figure S4. FTIR spectra of SPAN from experimental measurement and DFT predictions, revealing characteristic vibrational modes pertinent to its molecular structure.

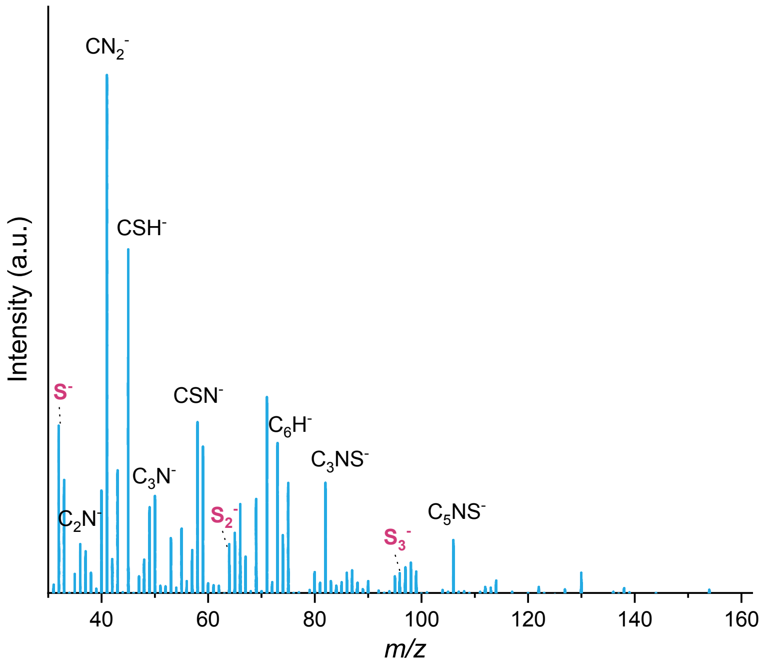

Figure S5. ToF-SIMS spectrum of pristine SPAN. Signal peaks from polysulfide are labeled purple.

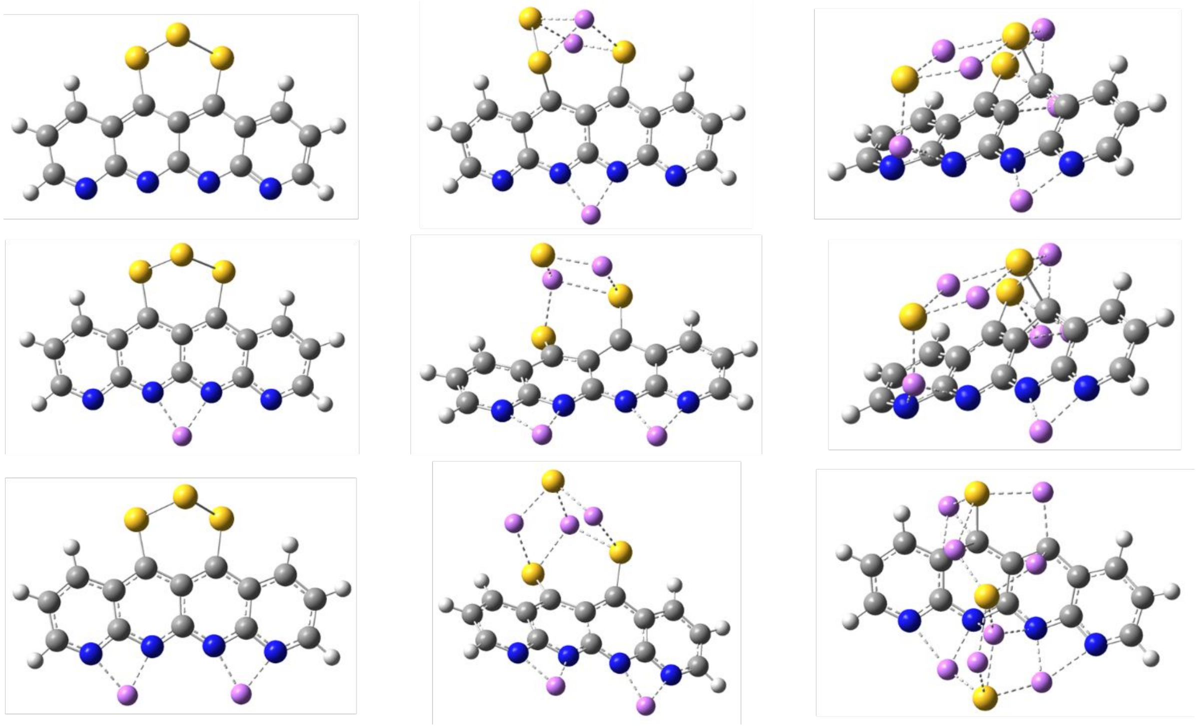

Figure S6. “Single-sided” SPAN molecular geometry variation due to lithiation. All structures have the lowest relative energies at the corresponding lithiation stages. Perspective rotations are employed to facilitate visualization of the atomic spatial positions. Additional structural and energy information is available upon request.

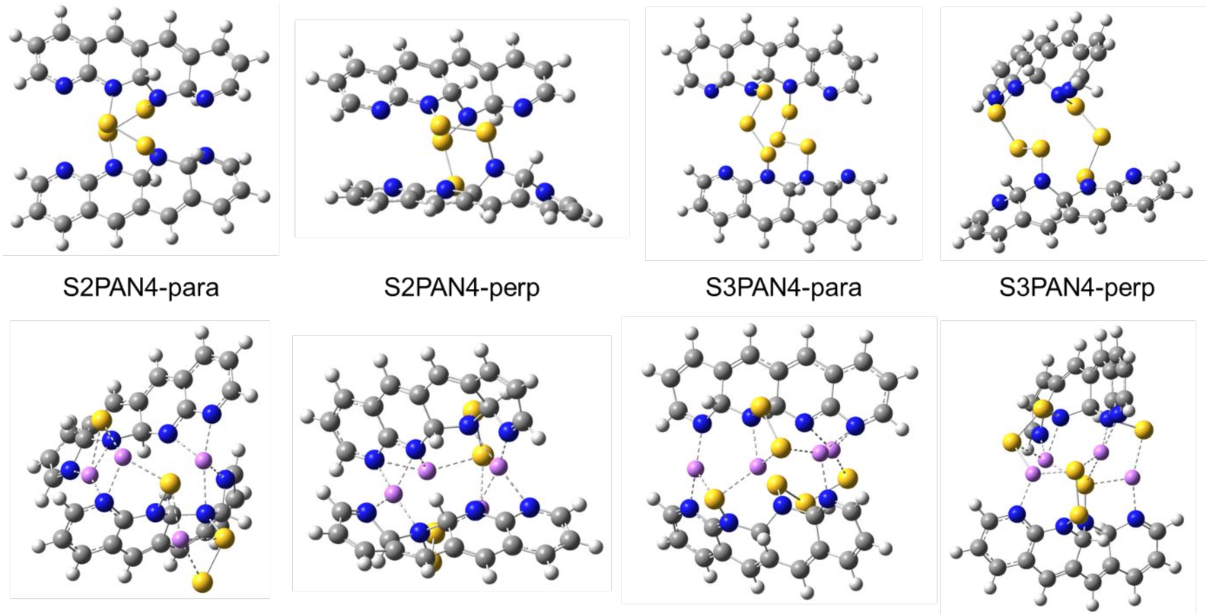

Figure S7. Unconstrained “bridging” SPAN molecular geometries before and after the forth lithiation. Perspective rotations are fostered to facilitate visualization of the atomic spatial positions. Additional structural and energy information is available upon request.

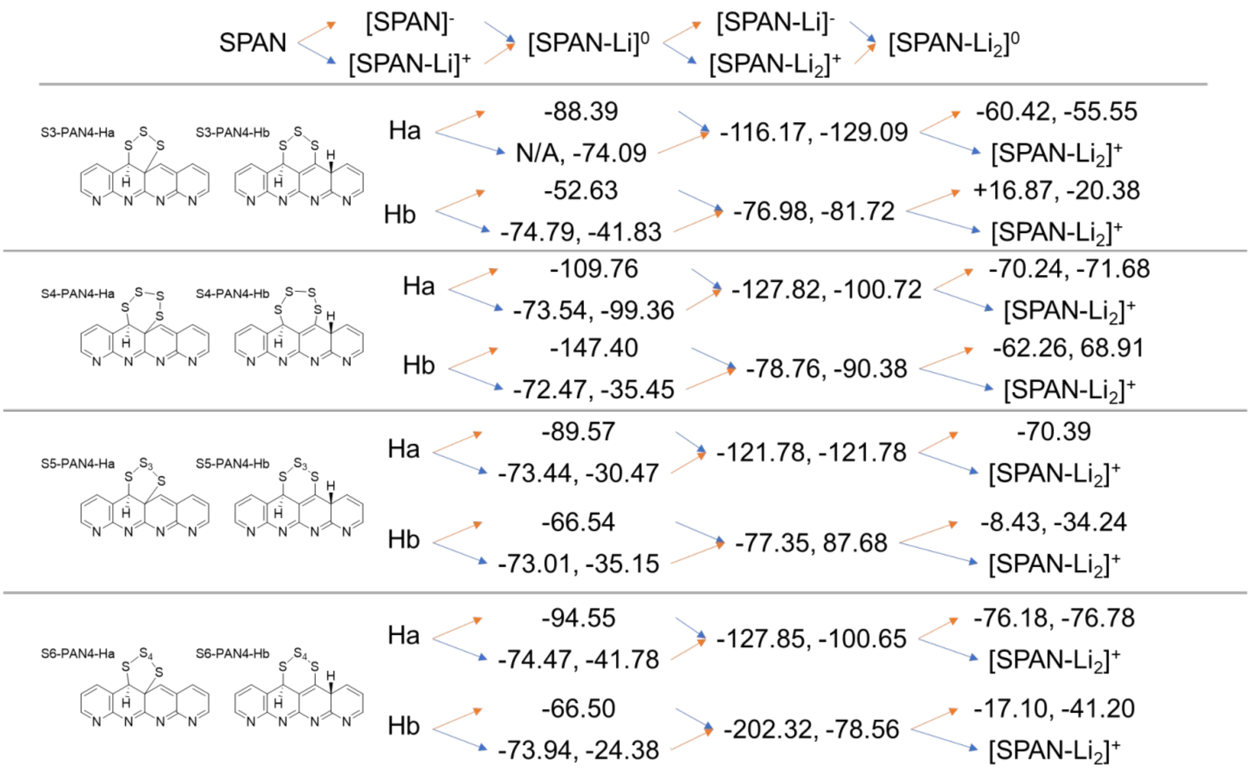

Figure S8. The electrochemical reduction free energies of S_x-C(4’) and S_x-C(3’/5’) structures. All units are in kcal/mol.

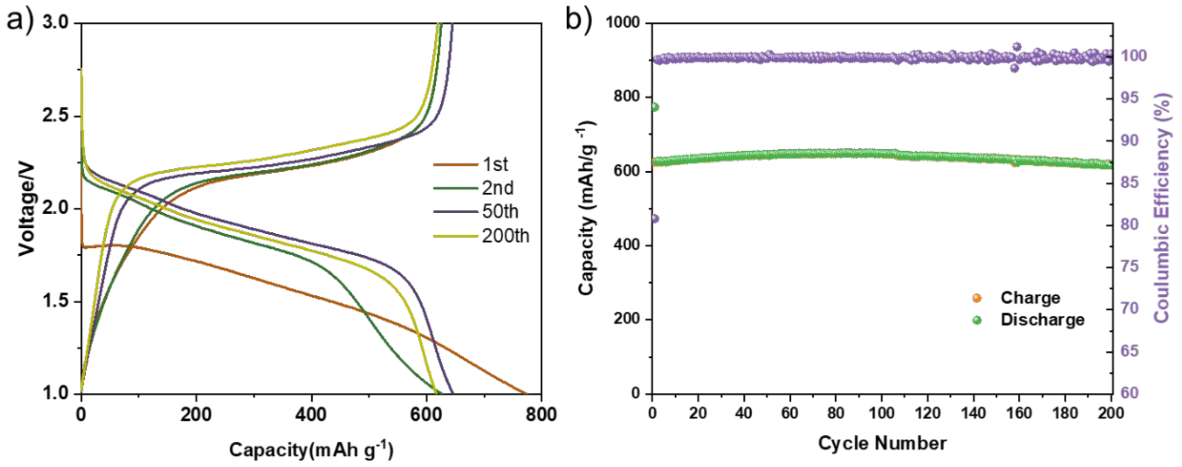

Figure S9. The discharge/charge profile (a) and the cycling stability (b) of a Li-SPAN battery tested at a 0.2 C rate with an areal loading of 2 mg·cm^{-2}. An irreversible capacity loss is noted immediately following the first discharge cycle. This loss is primarily attributed to a transformation within SPAN's chemical structure: several non-aromatic species are believed to be lost and converted into aromatic structures during this phase. Importantly, following this initial discharge and the associated structural reconfiguration of SPAN, the battery demonstrates a stable electrochemical behavior with no significant

capacity fade detected in subsequent cycles. This observation points towards a stabilization of the battery's electrochemical properties post the initial cycle, highlighting the reversible nature of the electrochemical reactions within SPAN after its initial conditioning.

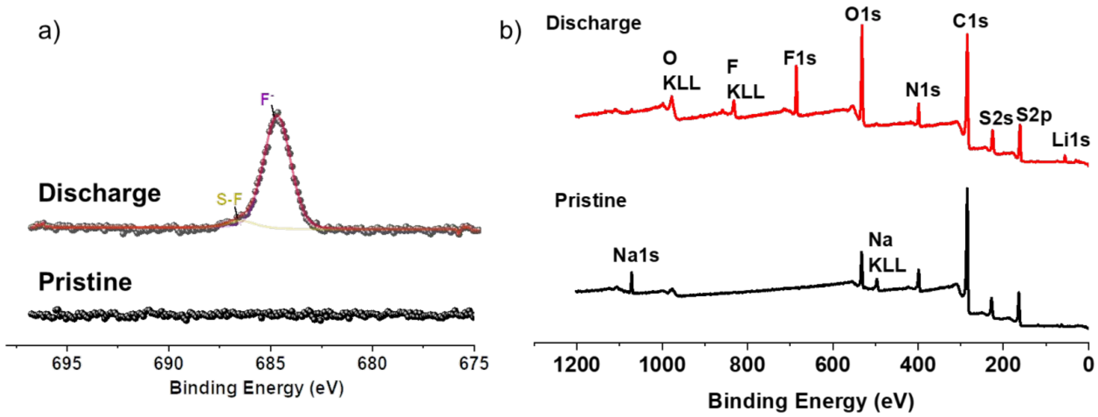

Figure S10. (a) High-resolution XPS analysis, the F1s spectra reveal the formation of two distinct species: fluoride ions (F^-) and sulfur-fluoride (S-F) bonds. (b) XPS survey spectra of a Li-SPAN battery before and after discharge. In the pristine state, the presence of sodium (Na) is detected within the spectrum, a result of incorporating carboxymethyl cellulose (CMC) as a binder, which contains sodium. Upon discharge, the emergence of a fluorine (F) peak is observed. This new peak is attributed to the decomposition of the lithium bis(fluorosulfonyl)imide (LiFSI) salt present in the electrolyte.

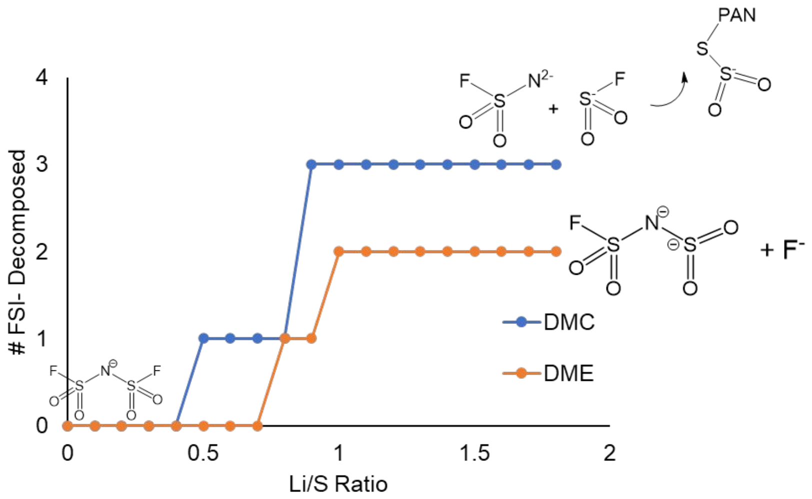

Figure S11. FSI^- decomposition pathways and fragments at the final stage of decomposition in DMC and DME obtained from AIMD simulations.

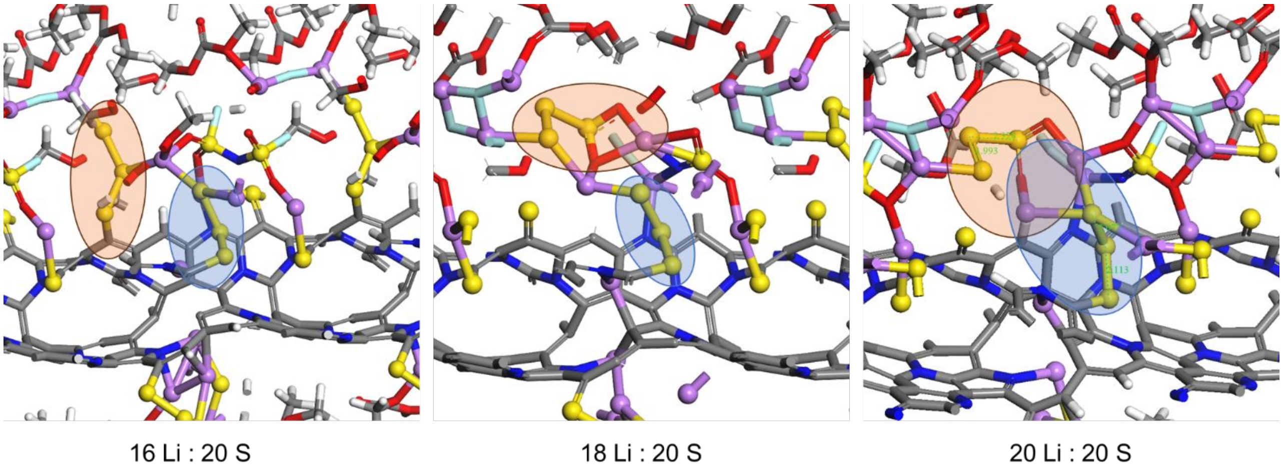

Figure S12. Illustration of SO_2^{2-} (generated from FSI degradation) bonding with PS through S_2-SPAN cleavage and S_2-SO_2 formation in the DMC-based electrolyte system. Snapshots from AIMD simulations.

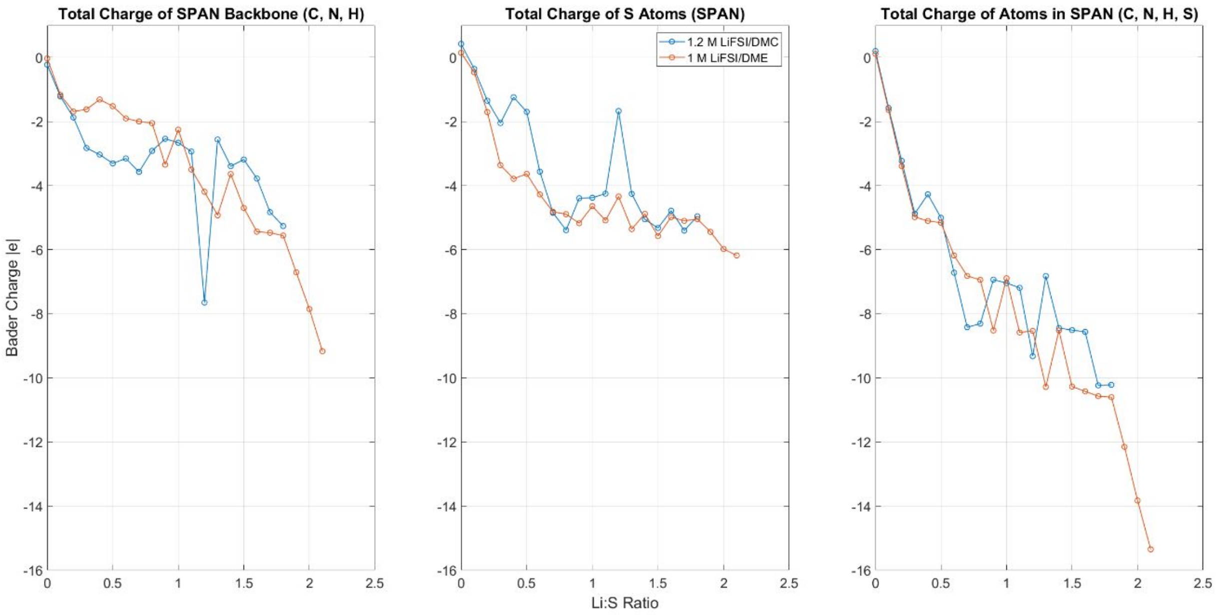

Figure S13. General charge evolution during SPAN lithiation in 1.2 M LiFSI/DMC (blue) and 1.0 M LiFSI/DME (red), respectively.

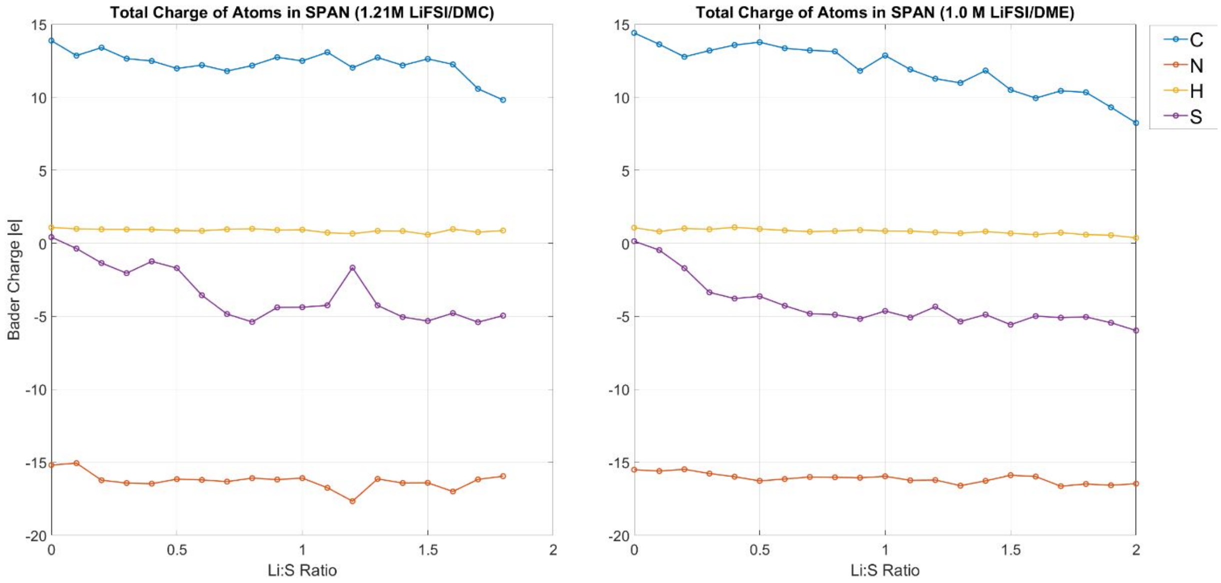

Figure S14. Charge evolution by element during SPAN lithiation in 1.2 M LiFSI/DMC (left) and 1.0 M LiFSI/DME (right), respectively.

Experimental Section: Synthesis and Characterization of Materials

Materials Synthesis

Commercially available reagents were procured from Sigma Aldrich and used as received unless specified otherwise. Sulfur and polyacrylonitrile (>150,000) were combined in a 4:1 weight ratio under an inert atmosphere and heated to 450 °C to synthesize SPAN.

Coin Cell Assembly and Electrochemical Testing

For the cathode fabrication, SPAN powder, carbon black (Super-P), and sodium carboxymethyl cellulose (CMC) binder were mixed in an 8:1:1 weight ratio in deionized water. The slurry was then spread onto aluminum foil and dried under vacuum at 80°C overnight. The resulting film was punched into 12 mm discs for use as the cathode with a mass loading of approximately 2.0 mg cm^{-2}.

The coin cells (2032 type) were assembled using the prepared SPAN cathode, lithium metal discs (500 µm thick, sourced from MTI), and a Celgard 2501 separator. The cells were filled with a sufficient amount of electrolyte consisting of 2 M Lithium bis(fluorosulfonyl)imide (LiFSI) in a solvent blend of 1,2-dimethoxyethane and bis(2,2,2-trifluoroethyl) ether (1:4 by weight, referred to as LDME). All cell assembly procedures were conducted in an argon-filled glove box. Electrochemical testing was conducted on a LAND battery tester (Wuhan, China) at room temperature, with galvanostatic charge/discharge cycles within a voltage window of 1–3 V vs. Li/Li^+ at a rate of C/5 (where 1C equals 600 mAh g^{-1} of SPAN).

Characterization Techniques

X-ray Photoelectron Spectroscopy (XPS): Measurements were conducted using an AXIS Supra instrument by Kratos Analytical with a monochromatized Al Kα radiation source (1486.7 eV). The samples were introduced from a nitrogen-filled glovebox directly connected to the XPS to maintain an ultra-high vacuum of 10^{-8} Torr. For surface cleaning, a 10 keV Ar^+ cluster ion beam was employed for 60 seconds. The C 1s peak at 284.6 eV served as a reference for charge correction, and spectra were processed using CasaXPS software.

Fourier-Transform Infrared Spectroscopy (FTIR): The FTIR spectra were acquired using a Nicolet 6700 spectrometer equipped with a Smart-iTR ATR attachment. The measurements were performed in ambient conditions.

Raman Spectroscopy: Washed with battery-grade DME to remove residual salts, the pristine and discharged SPAN electrodes were dried in an argon-filled glove box. To avoid air exposure, the samples were sealed on the sample side with a glass cover and on the substrate side with Kapton tape. The Raman spectra were recorded using a Renishaw inVia Raman Microscope with a 532 nm laser at 10% power to reduce potential thermal damage, employing a 50x objective across a spectral range of 100–2500 cm^{-1}.

Time-of-Flight Secondary Ion Mass Spectrometry (ToF-SIMS): Negative ion ToF-SIMS data were acquired using an IONTOF ToF-SIMS 5 spectrometer. Spectra/images were acquired using a 25 keV Bi^{3+} cluster ion source in the pulsed mode using high mass resolution mode. Data was acquired using a 100 micron x 100 micron spot at 256 x 256 pixels. The ion source was operated with a current of 0.14 pA to avoid saturation in the spectra. The primary ion dose per layer was 5.7×10^{11} ions/cm^2. An electron flood gun and argon flooding was also used for charge neutralization. Negative ion data were calibrated using the CH^-, OH^-, C_2H^- and C_4H^- peaks with calibration errors less than 20 ppm.
